# Hypothalamic Microglial Activation in Obesity: A Mini-Review

**DOI:** 10.3389/fnins.2018.00846

**Published:** 2018-11-15

**Authors:** Natália F. Mendes, Young-Bum Kim, Lício A. Velloso, Eliana P. Araújo

**Affiliations:** ^1^School of Nursing, State University of Campinas, Campinas, Brazil; ^2^Department of Medicine, Division of Endocrinology, Diabetes, and Metabolism – Beth Israel Deaconess Medical Center, Harvard Medical School, Boston, MA, United States; ^3^Laboratory of Cell Signaling, Obesity and Comorbidities Research Center, State University of Campinas, Campinas, Brazil; ^4^National Institute of Science and Technology on Neuroimmunomodulation, Rio de Janeiro, Brazil

**Keywords:** inflammation, chemokine, cytokine, brain, metabolism

## Abstract

Emerging data demonstrate that microglia activation plays a pivotal role in the development of hypothalamic inflammation in obesity. Early after the introduction of a high-fat diet, hypothalamic microglia undergo morphological, and functional changes in response to excessive dietary saturated fats. Initially the resident microglia are affected; however, as diet-induced obesity persists, bone marrow-derived myeloid cells gradually replace resident microglia. Genetic and pharmacological approaches aimed at dampening the inflammatory activity in the hypothalamus of experimental models of obesity have proven beneficial to correct the obese phenotype and improve metabolic abnormalities commonly associated with obesity. These approaches provide an experimental proof-of-concept that hypothalamic inflammation is central to the pathophysiology of obesity; understanding the details of the roles played by microglia in this process may help the development of preventive and therapeutic advances in the field. In this review, we discuss the potential mechanisms underlying hypothalamic microglial activation in high-fat induced obesity.

## Introduction

The mediobasal hypothalamus (MBH) is a key brain region involved in the central control of caloric intake and energy expenditure. Its function relies on a complex neuronal network that senses the energy status of the body and coordinates responses aimed at maintaining energetic homeostasis. First-order neurons in this network are located in the arcuate nucleus (ARC), sitting close to the median eminence (ME), which is richly irrigated by fenestrated capillaries that secure special permeability to the blood/spinal fluid interface (BSFI) (Rodríguez et al., 2010). The partially leaky ME-BSFI facilitates ARC neuronal and glial cells to rapidly and directly sense minimal changes in peripheral signals, such as hormones (e.g., leptin and insulin) and nutrients, essential to the regulation of whole-body energy homeostasis (Lam et al., 2005; Schaeffer et al., 2013; Timper and Brüning, 2017). Under physiological conditions, feeding leads to the activation of ARC proopiomelanocortin (POMC) and cocaine- and amphetamine-regulated transcript (CART) neurons resulting in suppressed food intake and increased energy expenditure. Conversely, fasting leads to the activation of neuropeptide Y (NPY) and agouti-related protein (AgRP) neurons, which antagonize the function of POMC neurons (Schwartz et al., 2000). Over time, the correct function of these neuronal subpopulations leads to body-mass stability. However, the excessive consumption of saturated fatty acids (SFAs) can disrupt the physiological pattern of food intake and energy expenditure control by triggering an inflammatory response in the hypothalamus, which affects the MBH at the functional and structural levels (De Souza et al., 2005; Velloso and Schwartz, 2011). Leptin resistance emerges as an early functional outcome of hypothalamic inflammation (Dragano et al., 2017; Pan and Myers, 2018), which can be overcome by reducing local inflammatory activity with genetic or pharmacological approaches, or simply by reducing the amount of SFAs in the diet. With time, chronic structural abnormalities can develop, such as changes in neuronal projections, gliosis, and eventually, neuronal death (Moraes et al., 2009; Dorfman and Thaler, 2015). The extension and chronicity of the structural and functional losses of the hypothalamus may explain the relapsing nature of obesity that can resist behavioral, pharmacological, and even surgical therapeutic measures. Thus, understanding the mechanisms involved in the evolution and persistence of hypothalamic inflammation may offer new insights into the development of strategies for the prevention and treatment of obesity.

Studies have shown that diet-induced inflammation initially and preferentially affects the MBH (Thaler et al., 2012). One of the particularities that may explain the anatomical specificity of this process is the nature of the ME-BSFI and the presence of tanycytes in this region, what makes the MBH more sensitive to dietary fats than other circumventricular organs, undergoing a rapid structural, and functional disorganization in mice fed a high-fat diet (HFD) (Ramalho et al., 2018). About the same time that structural and functional changes are occurring in the ME-BSFI, resident microglia undergo morphological and physiological alterations in the ARC region that neighbors the ME (Valdearcos et al., 2014). However, upon persistence of a HFD, bone marrow-derived myeloid cells (BMDM) migrate into the MBH, which is an important condition to secure chronic inflammation (Valdearcos et al., 2017). Approaches that inhibit the migration of BMDM cells to the MBH can reduce hypothalamic inflammation and attenuate diet-induced obesity (DIO). Despite the fact that most studies in this field were performed employing experimental models, there is evidence supporting the presence of inflammation and gliosis in the hypothalamus of obese humans as well (van de Sande-Lee et al., 2011; Thaler et al., 2012), further supporting hypothalamic inflammation as a potential pharmacological target for the obesity treatment. Here, we review the studies that provided progress in the characterization of abnormalities of MBH microglia in obesity.

## Dietary Fats Modify Hypothalamic Microglial Morphology, Distribution, and Function

Microglial cells are the most abundant brain innate immune cells and are relatively quiescent under baseline conditions. However, when exposed to injury signals, microglia undergo a rapid reaction characterized by morphological and functional changes aimed at enabling them to mount an inflammatory response against the potentially existing treat (Saijo and Glass, 2011).

Studies with rodents have shown that microglia of the MBH are sensitive to SFAs (lauric, palmitic, and stearic acids) (Valdearcos et al., 2014). In the hypothalamus of rodents, consumption of large portions of SFAs promotes a rapid microglial response characterized by the appearance of a number of prolonged processes emanating from a discreetly enlarged cell body (Baufeld et al., 2016). Initially, these morphological changes are restricted to the ARC region nearby the transition between the MBH and ME and are accompanied by increased expression of inflammatory cytokines and other markers of inflammation. The hypothalamic inflammatory response to SFAs occurs in a biphasic mode. During the first week of a HFD, the mRNA and protein levels of hypothalamic cytokines such as TNF-α, IL1-β, and IL-6 increase, as well as chemokines such as MCP-1 and fractalkine (Morari et al., 2014). Upon the persistence, for 2 weeks and longer, of the exposure to SFAs in diets that are particularly rich in stearate and palmitate, the levels of inflammatory markers decrease, only to return after 4 weeks, and then persist for as long as the content of SFAs in the diet is excessive (Thaler et al., 2012).

The biphasic nature of the inflammatory response in the hypothalamus of experimental models of obesity occurs similarly to the classical biphasic inflammatory response in other tissues. One reason behind the biphasic nature of obesity-associated hypothalamic inflammation is that the initial response is mounted by resident microglia; however, as dietary-fat consumption persists, bone marrow-derived myeloid cells gradually replace the MBH microglia (Valdearcos et al., 2017). Thus, at this point, it is important to understand the mechanisms that govern microglia activation, phenotype definition, and the recruitment of bone marrow-derived myeloid cells to the site of injury.

## Mechanisms of Hypothalamic Microglia Activation in Obesity

Similar to the resident macrophages of tissues outside the brain, microglia of rodents are capable of sensing and responding to the presence of pathogen-associated molecular patterns (PAMPs) (Kigerl et al., 2014). The systemic injection of a single high-dose of lipopolysaccharide (LPS) promotes activation-like changes in the morphology of hypothalamic microglia, which is detected as early as 8 h after injection, peaking after 24 h and recovering after 7 days (Buttini et al., 1996). This is accompanied by increased expression of MHC-I and inflammatory cytokines (TNF-α, IL-1, and IL-6), and also by the activation of COX-1 and NF-κB (García-Bueno et al., 2009). Interestingly, the same macrophage receptor that is activated in response to LPS, TLR4, can be activated in response to SFAs (Huang et al., 2012).

Under baseline conditions, TLR4 is expressed at low levels in the brain, including the hypothalamus of rodents; however, its expression increases considerably when rodents are fed a HFD (Milanski et al., 2009). In other brain regions, TLR4 expression is involved in medical conditions such as autoimmunity (Kerfoot et al., 2004), HIV infection (Cheung et al., 2017) and Alzheimer’s disease (Ledo et al., 2016), illustrating its pleiotropic roles in brain immunity and defense. A detailed screening of dietary fats that could potentially promote the activation of hypothalamic TLR4 identified long-chain SFAs as the most potent inducers of its association with MyD88, resulting in the activation of NF-κB and JNK and leading to endoplasmic reticulum stress (Zhang et al., 2008). When dietary SFAs are substituted by similar amounts of unsaturated fatty acids (flax seed oil and olive oil), the hypothalamic inflammation is dampened in parallel to the reduced activity of JNK and NF-κB (Cintra et al., 2012). The pharmacological and the genetic inhibition of TLR4 reduce diet-induced hypothalamic inflammation and protect rodents from DIO and glucose intolerance (Milanski et al., 2012). Importantly, contact with large portions of SFAs, and not the presence of obesity *per se*, is required to induce hypothalamic microglial activation; this is evidenced by the fact that genetically obese mice, such as, ob/ob (leptin-deficient), db/db (leptin receptor-deficient) and MC4R-KO, do not present hypothalamic microglia activation when they are fed on chow, despite the evidence that even on this diet they become obese (Gao et al., 2014).

Cell culture approaches were used to investigate additional details involving the activation of microglia by fatty acids. Palmitate and stearate are each capable of inducing a rapid activation of BV2 microglial cells line through a mechanism dependent on TLR4 (Wang et al., 2012). Reactive morphological changes and increased production of inflammatory cytokines, nitric oxide, and reactive oxygen species (ROS) characterize the activation of microglia (Wang et al., 2012). In addition, upon exposure to palmitate, another microglial cell line, THP-1, is capable of producing inflammatory cytokines and inducing neurotoxicity (Little et al., 2012).

As a proof-of-concept for the central implication of microglia in the development of diet-induced hypothalamic inflammation, Valdearcos et al. (2014) used distinct strategies to modify the number of microglia in the hypothalamus. When microglia is increased, the intensity of hypothalamic inflammation induced by SFAs is boosted; conversely, when MBH microglia is reduced hypothalamic inflammation is abrogated. Importantly, a positive relationship exists between the number of hypothalamic microglia and the obese phenotype (Baufeld et al., 2016).

In addition to the direct effects of dietary fats to induce hypothalamic inflammation and microglial activation, some studies have shown that neural afferents from the gut can also trigger an inflammatory response in the hypothalamus (Mulders et al., 2018). The gut inflammatory signals resulting from the consumption of HFD can act through vagal connections to the nodose ganglion to increase hypothalamic microglial cells and trigger inflammation in a mechanism that can be inhibited by ghrelin (Waise et al., 2015). Furthermore, in at least one study, the gut microbial landscape changes promoted by consumption of a HFD was shown to play a role in the development of diet-induced hypothalamic microgliosis (Zhuang et al., 2017).

It is worthwhile mentioning that brain regions other than hypothalamus can also present microglial activation in response to excessive consumption of dietary fats (Erion et al., 2014; Spencer et al., 2017). However, differently of the hypothalamus, microgliosis in other regions is a late event, reinforcing the current concept that hypothalamus is the first brain region affected in response to the consumption of large amounts of dietary fats (Thaler et al., 2012; Carraro et al., 2018). Particularly, in the hippocampus, diet-induced microgliosis has been identified in both human and experimental models of Alzheimer’s disease and provides a potential mechanistic link between obesity/type 2 diabetes and the accelerated cognitive and memory decline that is present in this highly prevalent medical condition (Cope et al., 2018).

## Intracellular Signaling Targets for Reducing Microglia Inflammatory Activation

Microglial cells express triggering receptor of myeloid cell-2 (TREM2), which is an important negative regulator of NF-κB. *In vitro* studies showed that TREM2 overexpression inhibits neuroinflammation by down-regulating PI3K/AKT and NF-κB signaling in BV2 microglia (Zhong et al., 2017; Li et al., 2018). Curiously, LPS downregulates the expression of TREM2 through the activation of JNK and NF-κB signaling pathways, resulting in a pro-inflammatory vicious cycle (Zhong et al., 2017).

Quercetin, a polyphenolic flavonoid found in many dietary sources, is a molecule studied due to its anti-inflammatory functional properties and also reduces inflammatory responses through inhibition of NF-κB activity. Quercetin can suppress the lipid content inside the palmitic acid-treated microglia through heme oxygenase (HO-1) induction (Kang et al., 2013; Yang et al., 2017a). HO-1 is an antioxidant enzyme that plays a key role in defense mechanisms against oxidative damage and inflammation. In addition, quercetin suppresses LPS-induced nitric oxide (NO) production and inducible NO synthase (iNOS) expression in BV2 cells (Kang et al., 2013).

Alpha-viniferin belongs to the same polyphenol group as quercetin and has anticancer and anti-inflammatory activity. In LPS-BV2 treated cells, α-viniferin suppresses the expression of the proinflammatory genes iNOS and COX-2 in the early stage of inflammation by inhibiting AKT/PI3K-dependent NF-κB activation and inhibits the production of proinflammatory mediators NO and PGE2 in the late stage by stimulating the Nrf2-mediated HO-1 signaling pathway (Dilshara et al., 2014). It is important to highlight that although many *in vivo* and *in vitro* studies have already shown these anti-inflammatory actions, the bioactive effects and bioavailability of these compounds in humans still need to be further investigated (Shanely et al., 2010; Pfeuffer et al., 2013; Lee et al., 2016).

Researchers have also studied other nutrients with functional properties in the context of HFD-induced microglial activation through modulation of NF-κB activity. Eicosapentaenoic acid (EPA) and docosahexaenoic acid (DHA) are the major ω-3 PUFAs of fish oil and have exhibited many anti-inflammatory effects in HFD-induced hypothalamic inflammation (Cintra et al., 2012; Nascimento et al., 2016). In the MG6 and BV2 microglial cell lines, EPA and DHA each suppressed the production of pro-inflammatory cytokines TNF-α and IL-6 throughout the inhibition of NF-κB activity and the activation of SIRT1 signaling (Inoue et al., 2017).

A few studies also investigated the potential involvement of ROS production as a mechanism of transducing inflammatory signals in microglia. Microglial inflammatory-response activation increases the production of oxidative-stress products. It is known that mitochondria constitute the major source of ROS generation in activated microglia (Park et al., 2015). ROS are capable of modifying microglial pro-inflammatory gene expression by modulating kinase cascades and activating some transcription factors, including MAPK and NF-κB (Pawate et al., 2004). LPS-treated BV2 cells show increased ROS production through NADPH oxidase, which raises the expression of TNF-α, activating NF-κB (Sumi et al., 2010). Otherwise, when these cells are treated with a NADPH oxidase inhibitor, ROS production decreases (Mander et al., 2006).

Thus, NF-κB and JNK signaling systems play central roles in the transduction of inflammatory signals in microglia. They can be either activated or inhibited by various nutrients, which make them important targets for approaches aimed at dampening HFD-induced hypothalamic inflammation. It is important to consider that due to the complex nature of human diet as compared to experimental diets offered to rodents, further clinical studies will be required in order to confirm the inflammatory nature of the hypothalamic damage in human obesity.

## Microglia Polarization

Microglial cells are able to sense changes in the surrounding environment and become activated to either a pro-inflammatory (M1) or an anti-inflammatory (M2) phenotype (Kettenmann et al., 2011; Tang and Le, 2016). Polarized microglia are distinguished from polarized peripheral macrophages by protein expression, phagocytic capacity, and response to injuries (Durafourt et al., 2012; Girard et al., 2013). Considering that HFD leads to the recruitment of peripheral myeloid cells to the MBH, it is important to understand if some differences exist in the molecular mechanisms of polarization between microglial and infiltrated peripheral myeloid cells in the central nervous system (CNS).

Microglial cell polarization has an energetic demand and, thus, depends on uncoupling protein 2 (UCP2) activity, which is regulated by fatty-acid-binding proteins (FABP) (Hotamisligil and Bernlohr, 2015). Mice lacking FABP4 in the hypothalamus have increased UCP2 expression and reduced expression of TNF-α and ionized calcium-binding adapter molecule 1 (Iba1, a marker of microglial activation (Duffy et al., 2017). Pharmacological inhibition of FABP is an interesting therapeutic approach to reducing saturated-fat-induced pro-inflammatory response because it increases UCP2 expression. In addition, the inhibition of hypothalamic UCP2 increases the inflammatory damage inflicted by TNF-α, which places UCP2 as an important component in the polarization machinery of microglia (Degasperi et al., 2008).

Studies have identified a number of compounds that can reduce inflammation in the CNS by suppressing microglia M1 phenotype (Zhou et al., 2014; Orihuela et al., 2016). Conversely, only a few compounds, such as interleukin-4 and interleukin-13, can promote the polarization of microglia to the M2-like anti-inflammatory phenotype (Zhou et al., 2014). In this context, promoting an M1-M2 switch could be an interesting approach to attenuate inflammation of the neural tissue (Hu et al., 2015; Xu et al., 2015). In line, a recent study showed that betulinic acid (BA), a naturally pentacyclic triterpenoid with anti-inflammatory properties, promotes M2 polarization and inhibits M1 polarization in LPS-stimulated BV2 microglial cells and in the cerebral cortex of LPS-injected mice through calmodulin-dependent protein kinase kinase β (CaMKKβ)-dependent AMP-activated protein kinase (AMPK) activation (Li et al., 2018). In another study, Xu et al. (2015) showed that telmisartan, a potent angiotensin II receptor blocker, also prevents LPS-induced microglia activation throughout M2 microglia polarization through CaMKKβ-dependent AMPK activation. Another mechanism that leads to microglia M1/M2 polarization depends on PGC-1α inhibition, which can be induced by LPS. Some compounds, like resveratrol, a natural polyphenol with anti-inflammatory effects, has beneficial effects on microglia activation throughout PGC-1α activation, inhibiting the M1-like phenotype while stimulating the M2-like phenotype (Yang et al., 2017b).

Although a microglial switch offers a promising avenue for reducing HFD-induced microglial inflammation, further characterization of microglial phenotypic behavior, especially its temporal and spatial evolution, is critical in electing potential therapeutic approaches. Furthermore, it is important to consider that microglial polarization is a dynamic and natural reaction to saturated-fat overconsumption and its complete blockage could impair microglial activity in resolving inflammation.

## The Recruitment of Peripheral Myeloid Cells to the Hypothalamus in Dio

Peripheral myeloid cells are recruited to MBH in mice fed a HFD and contribute to the development of obesity-associated hypothalamic microgliosis (Morari et al., 2014; Valdearcos et al., 2017). The fenestrated nature of the BSFI surrounding the MBH is regarded as an important factor involved in this process (Ramalho et al., 2018). Numerous studies showed that bone-marrow-derived microglia (BMDM) can infiltrate the brain parenchyma in distinct types of brain injury (Ajami et al., 2007; Davoust et al., 2008; Prinz and Mildner, 2011). Valdearcos et al. (2017) demonstrated that myeloid cells infiltrating the MBH in mice fed a HFD expressed neither typical microglial markers nor markers indicative of a bloodborne origin. Thus, it was initially suspected that these cells could represent a non-microglial population of brain-resident myeloid cells.

Under normal conditions, BMDM does not renew microglial cells, but under certain circumstances in which cells present in systemic circulation cross the BSFI, they can undergo differentiation into microglia. Thus, the adult bone marrow contains a subpopulation of cells displaying a microglial differentiation potential that can be mobilized promptly at any time (Davoust et al., 2008); however, which factors induce this specific type of monocyte chemotaxis into the MBH in the context of dietary excess is currently under investigation.

Fractalkine (CX3CL1), a chemokine that signals through the CX3CR1 receptor, is involved in this earlier activation of hypothalamic inflammation in HFD-fed mice (Morari et al., 2014). Another chemokine axis, MCP-1/CCR2, has also been described as an important potential mediator of the migration of monocytes into affected areas in brain diseases (Deshmane et al., 2009). Under chronic stress, e.g., a long period of high-fat feeding, the BMDM are recruited to the paraventricular nucleus of the hypothalamus (PVN) throughout, after the activation of the MCP-1/CCR2 axis (Ataka et al., 2013). In this study, the authors blocked the recruitment by peripheral administration of a CCR2 antagonist, which resulted in a reduction of local inflammation, providing evidence of an important role of the MCP-1/CCR2 axis in diet-induced hypothalamic inflammation.

Although the origin and identity of myeloid cells that migrate to the MBH after HFD consumption are unknown, these cells displayed morphological features of resident microglia (Valdearcos et al., 2017). The BMDM, as resident microglia, can be clearly distinguished from other CNS-infiltrating macrophages, based on its CD45 expression (Priller et al., 2001). In addition, BMDM can proliferate in the neural tissue when inflammation is induced, whereas infiltrating macrophages do not (Wirenfeldt et al., 2007).

Another important question still unanswered refers to the mechanism that induces the recruitment of BMDM into the peripheral circulation because it precedes recruitment to the CNS. SDF-1/CXCR4 interaction is required to recruit BMDM out of the bone marrow toward systemic circulation (Katayama et al., 2006). SDF-1 expression, in turn, is regulated by brain stimuli through sympathetic pathways, throughout β3-adrenergic receptors on bone marrow stromal cells (Lilly et al., 2011). Thus, it is possible that the signal that triggers the whole process is mediated by autonomic connections between the hypothalamus and the bone marrow; however, further studies are needed to clarify this important issue.

**FIGURE 1:**
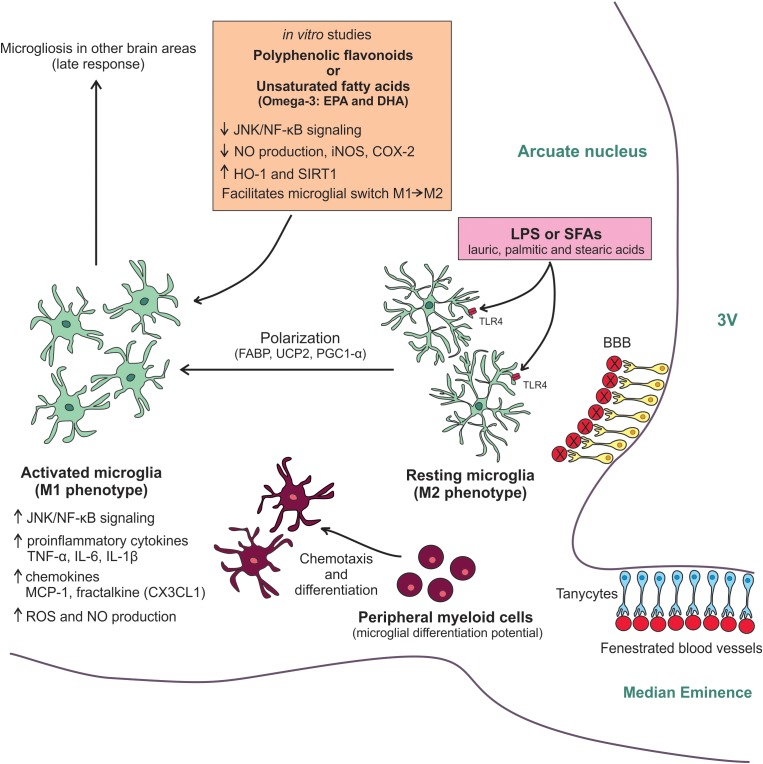
Molecular mechanisms involved in the HFD-induced hypothalamic microgliosis. Diet-induced inflammation initially affects the MBH. Due to its anatomic location, microglial cells of arcuate nucleus are sensitive to LPS and SFAs from the diet. Both molecules activate TLR4 in the microglia membrane resulting in the switching to M1-like pro-inflammatory phenotype. This M2/M1 polarization has an energetic demand and depends on UCP2 activity, which is regulated by FABP, and on PGC-1α activation. The microglia activation results in an increased JNK/NF-kB activity, raised expression of pro-inflammatory cytokines and chemokines, as well as increased ROS and NO production. With the persistence of the high-fat feeding, peripheral myeloid cells are recruited to the neural tissue. These cells have a microglial differentiation potential and can quickly differentiate in activated cells helping resident microglia smother the hypothalamic inflammatory response. The chemotaxis of these peripheral cells is mainly controlled by MCP-1/CCR2 axis and fractalkine. As a late response, the microgliosis can also affect other non-hypothalamic brains areas. Some anti-inflammatory and antioxidants molecules, such as polyphenolic flavonoids and unsaturated fatty acids, have been tested *in vitro* for attenuating the microglial activation. The main benefits found in these studies were the reduction of JNK/NF-kB activity, decreased NO production, as well as iNOS and COX-2 expression, and increased HO-1 and SIRT-1 expression. Besides, these molecules also seem to facilitate the microglial switch M1/M2, reducing the inflammatory response. Further studies are still needed to further test these mechanisms in experimental models.

In conclusion, the current data are reshaping the concept that resident microglia constitutes a stable brain-cell population that is not affected by the recruitment of BMDM (Matsumoto and Fujiwara, 1987; Lassmann et al., 1993). Certain types of brain injury, including the damage caused in the hypothalamus by the excessive consumption of SFAs, can recruit BMDM, which seem to be an important event to modulate the inflammatory response. Particularly, after consumption of a HFD, it seems the recruitment of peripheral myeloid cells to the hypothalamus occurs in an attempt to help the resident microglia smother the inflammatory response. As this response happens at the beginning of HFD overconsumption, exploring which mechanisms are involved and how it alters hypothalamic neurocircuits involved in metabolic control represents a new frontier for proposing a viable therapeutic approach before the damage is established. Figure 1 provides a summarized view of the mechanisms involved in the regulation of hypothalamic microglia in obesity.

## Outstanding Questions

(1) How do different subsets of microglia participate in the HFD-induced hypothalamic inflammation?
(2) Could microglia from other brain areas also migrate to the MBH contributing to the local inflammation?
(3) Is the recruitment of BMDM triggered by hypothalamic outputs? If so, which mechanisms are involved?
(4) Is there inflammation and microglial activation in the hypothalamus of obese humans? If so, is there any possibility of pharmacologically targeting this inflammation to treat human obesity?

## Author Contributions

All authors wrote the paper and approved the final version of the manuscript.

## Conflict of Interest Statement

The authors declare that the research was conducted in the absence of any commercial or financial relationships that could be construed as a potential conflict of interest.
